# An overview of antipsychotic drug prescribing trends (initiation/prevalence) in UK primary care from 1995 to 2018: analysis of electronic health records from over 790 general practices

**DOI:** 10.1192/bjo.2025.10815

**Published:** 2025-08-14

**Authors:** Siti Watiqah Samsuddin, Claudia Cooper, Joseph Hayes, Juan Carlos Bazo-Alvarez, Patricia Schartau, Irene Petersen

**Affiliations:** Department of Primary Care and Population Health, Institute of Epidemiology and Health Care, University College London, UK; Centre for Psychiatry and Mental Health, Wolfson Institute of Population Health, Queen Mary University of London, UK; Division of Psychiatry, Faculty of Brain Sciences, University College London, UK

**Keywords:** Antipsychotic, electronic health records, primary care, pharmacoepidemiology

## Abstract

**Background:**

Initially prescribed for schizophrenia and psychosis, antipsychotics are increasingly prescribed for other indications. Since the late 1990s, prescribing shifted from first-generation to second-generation antipsychotics.

**Aims:**

To examine overall initiation and prevalence of antipsychotic drug prescribing in UK primary care from 1995 to 2018, stratified by gender.

**Method:**

Cohort studies using UK anonymised electronic primary care data from IQVIA Medical Research Data, including over 790 general practices and registered individuals aged 18–99 years.

**Results:**

Antipsychotic drug initiation was stable in the late 1990s, at 6–7/1000 person-years at risk (PYAR) in men and 9–11/1000 PYAR in women. From 2001, initiation declined, stabilising from 2005 onward at 4/1000 PYAR in men and 4–5/1000 PYAR in women. Prevalence remained consistent from 1995 to 2018: 12/1000 in men and 14/1000 in women by 2018. Initiation and prevalence were higher in women than men, but increased with age in both genders: (18–39 *v*. 80–99 years; incidence rate ratio (IRR) 4.85, 95% CI 4.75–4.95 in men; IRR 5.90, 95% CI 5.78–6.02 in women; prevalence rate ratio (PRR) 2.22, 95% CI 2.19–2.25 in men; PRR 4.28, 95% CI 4.24–4.33 in women). Initiation and prevalence were greater in individuals with greater socioeconomic deprivation (Townsend score of 5 *v*. 1; IRR 2.69, 95% CI 2.64–2.75 in men; IRR 2.19, 95% CI 2.15–2.24 in women; PRR 3.87, 95% CI 3.82–3.92 in men; PRR 2.80, 95% CI 2.77–2.83 in women).

**Conclusions:**

Antipsychotic drug initiation decreased after 2001, stabilising from 2005 onward. Prevalence remained relatively consistent throughout the study period. Women had higher initiation and prevalence than men. However, both genders showed increased prescribing with age and socioeconomic deprivation.

Antipsychotic medication remains the key pharmacological treatment of schizophrenia and psychosis.^1–3^ However, it is also used for several other indications, including bipolar disorder, depression, and behavioural and psychological symptoms of dementia.^4^ Over the past three decades, we have seen a change in types of antipsychotics being prescribed in the UK. In the early 1990s, first-generation antipsychotics (FGAs) were predominantly prescribed, which were then gradually replaced by second-generation antipsychotics (SGAs) in the 2000s.^4–10^ In 2002, The UK National Institute for Health and Care Excellence (NICE) introduced their first guideline on treatment of schizophrenia; the guideline recommended the extensive use of SGAs for individuals with schizophrenia, based on their fewer side-effects profile compared with FGAs.^11^ In the same time period, several randomised controlled trials were conducted on SGAs; several of these trials reported safety concerns on the use of SGAs, particularly risperidone and olanzapine, and increased rate of cerebrovascular events in people with dementia.^12,13^ Following this, the UK’s Committee of Safety on Medicines had released recommendation on the use of SGAs for individuals with dementia in 2004.^14^ Previous studies have reported higher prevalence of all-type dementia and Alzheimer’s disease among women than men.^15^ Thus, our key hypothesis is that antipsychotic drug prescribing, especially SGAs, was more common among older women by 2018. Several studies using primary care data-sets have described prescribing trends in shorter or longer periods of time of up to 2011.^4,5^ To our knowledge, our study is the first to provide an overview of the initiation and prevalence, with greater focus on stratification by gender, covering the period between 1995 and 2018. Our analyses are based on prescribing data from the primary care electronic records, therefore provide a wider representation of the use of antipsychotic treatment in the UK population irrespective of underlying indications.

## Method

### Study design

A series of cohort studies from 1 January 1995 to 31 December 2018.

### Setting

In the UK, the first point of contact is often primary care providers, including general practitioners (GPs). Subsequently, a primary care provider can refer patients to secondary care for emergency, elective and mental healthcare, or tertiary care for more advanced specialist treatments. Although treatment for severe mental illness such as schizophrenia is often initiated by specialists in secondary care, most treatments, excluding antipsychotic drug injections and oral clozapine, will often be continued by GPs. Each medication to be prescribed on the primary care system will be linked to the Anatomical Therapeutic Classification (ATC) and British National Formulary (BNF) codes, which reflect the standard specified by the NHS England under the NHS Dictionary of Medicines and Devices.^16^

### Data source

We used anonymised electronic primary care records from IQVIA Medical Research Data (IMRD), incorporating data from THIN, a Cegedim database. IMRD comprises data extracted from over 790 general practices across the UK, covering approximately 6% of the national population, and it is largely representative of the UK population in terms of demographics and geographic distribution.^17,18^ All practices were using the Vision software for their patient management. IMRD contains individual-level data including gender, year of birth, date of death, diagnoses and dates of diagnoses, and social deprivation (Townsend score). Also incorporated in the database is information such as individuals’ date of registration with the general practice, date of transfer from the practice and prescribed medications, including generic drug name, formulation and the dates prescriptions were issued.

The quality of the data used were identified using the following two markers: acceptable mortality rate (AMR) and acceptable computer usage (ACU). ACU is defined as the year in which a general practice was continuously recording on average at least two therapy records, one medical record and one additional health data record per patient per year.^19^ This indicates that a practice has moved from paper-based to computer-based management of patient care. AMR is defined as the date when mortality recording of a practice reached that of the general UK population, after considering the age and gender formation of the practice population.^20^

Townsend scores were used to represent individuals’ socioeconomic background, where a Townsend score of 1 indicates those from the least deprived background, and 5 indicates those from the most deprived background. Townsend scores are derived from census data based on calculations on four indicators of deprivation: non-home ownership, non-car ownership, unemployment and overcrowding.^21^

### Participants

All individuals aged from 18 to 99 years who were registered with participating GPs at any point during the study period were eligible for the study.

### Follow-up period

Individuals were followed from the latest date of the following: (a) the start of the study period, 1 January 1995; (b) individual turned 18 years old; (c) registration with the practice contributing data to the database or (d) when practices start contributing data at high quality. The follow-up period ended at the earliest date of the following: (a) the end of the study period, 31 December 2018; (b) date of the individual’s death; (c) individual left the practice contributing data to the database or (d) for the study of initiation, the date of the first record of an antipsychotic prescription. We did not have access to data beyond 2018.

### Definition of initiation and prevalence

Some individuals may be prescribed antipsychotic several times in their lives. We considered an individual to be initiated a new antipsychotic treatment if there were more than 12 months between two treatment episodes. Prevalence was defined as individuals who were prescribed at least one antipsychotic per calendar year during the study period. For example, if an individual was prescribed an antipsychotic from February to July 2002, and then again from June to October 2008, the individual would be considered to have contributed to the prevalence data once in 2002 and again in 2008. The list of antipsychotic drugs investigated in our study is listed in Supplementary Appendix 1 available at https://doi.org/10.1192/bjo.2025.10815.

Unadjusted initiation rate was estimated per 1000 person-years at risk (PYAR). Unadjusted prevalence was calculated by dividing the total number of individuals prescribed at least one antipsychotic medication (numerator) by the total number of individuals registered with the general practice in the given year (denominator). All initiation and prevalence estimates were stratified by gender and reported by calendar year, age groups (18–39, 40–59, 60–79 and 80–99 years) and socioeconomic deprivation level (Townsend quintile 1 being least deprived and 5 being most deprived).

When calculating prevalence, we only included the time periods when individuals were contributing data for the full calendar year. For example, if an individual’s index date and end date were 17 July 1997 and 10 June 2003, respectively, data for that the specified individual would have included data from 1 January 1998 until 31 December 2002.

### Statistical analysis

When describing the demographics of the study participants, we chose individuals who were included in the study in 1995 and 2018, thus representing the start and end of our study, respectively. Further information is available in Supplementary Appendix 2.

Multivariable Poisson regression models including robust standard errors were fitted to determine changes over the calendar year in initiation and prevalence, reported as incidence rate ratios (IRRs) and prevalence rate ratios (PRRs), respectively (reference year 1995). Reported IRRs and PRRs for the calendar year were stratified by gender and adjusted for age and socioeconomic deprivation level. We also reported IRR and PRR for age bands (reference: 18–39 years old) and socioeconomic deprivation (reference: least deprived, indicated as Townsend score of 1) when adjusting for other respective variables. Log likelihood ratio tests were used to examine interactions between gender and the other variables.

**Fig. 1:**
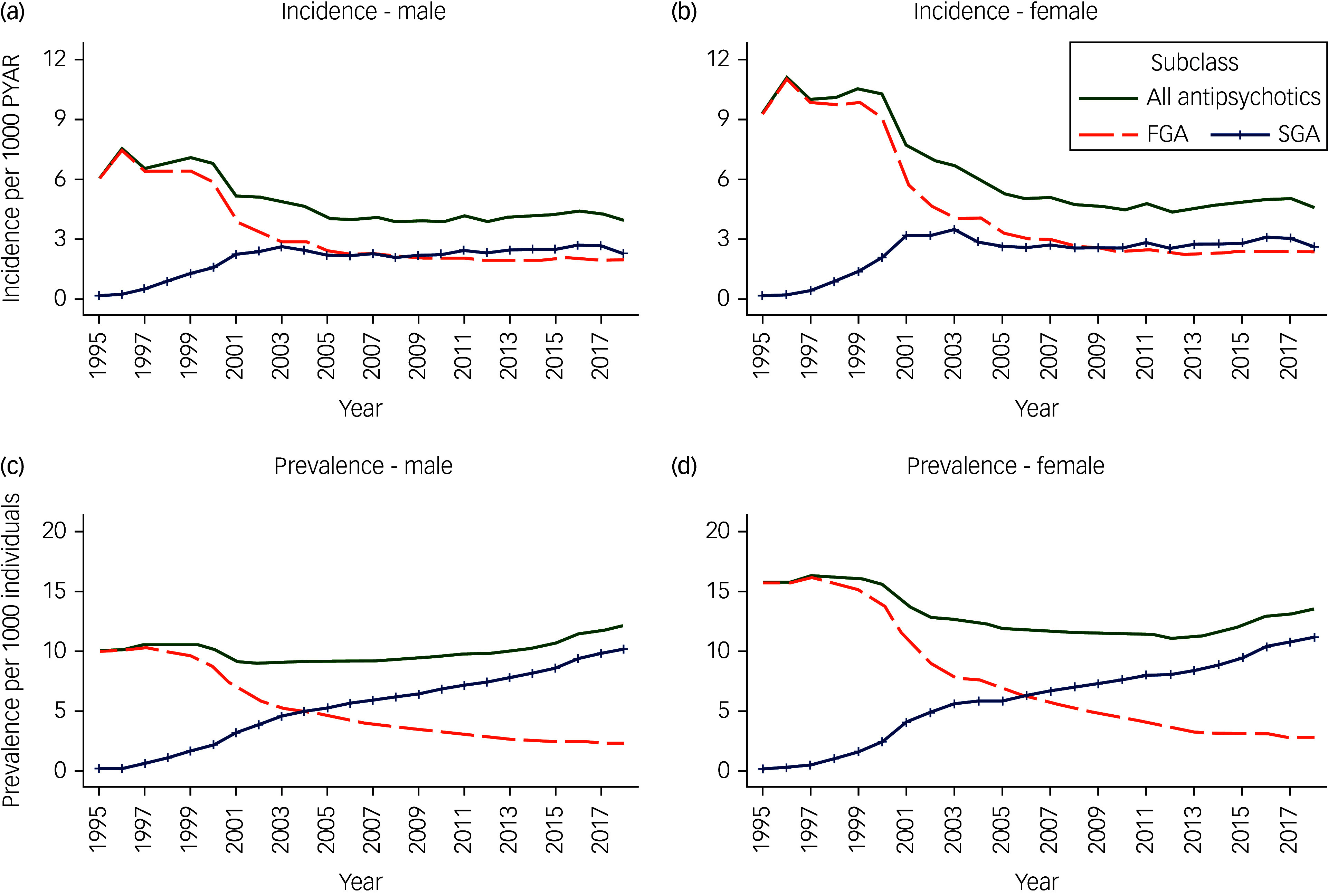
Unadjusted initiation of antipsychotic drug prescribing per 1000 person-years at risk (PYAR) (a, b), and unadjusted prevalence of antipsychotic drug prescribing per 1000 individuals (c, d) from 1995 to 2018 by subclasses, stratified by gender. FGA, first-generation antipsychotic; SGA, second-generation antipsychotic.

**Fig. 2:**
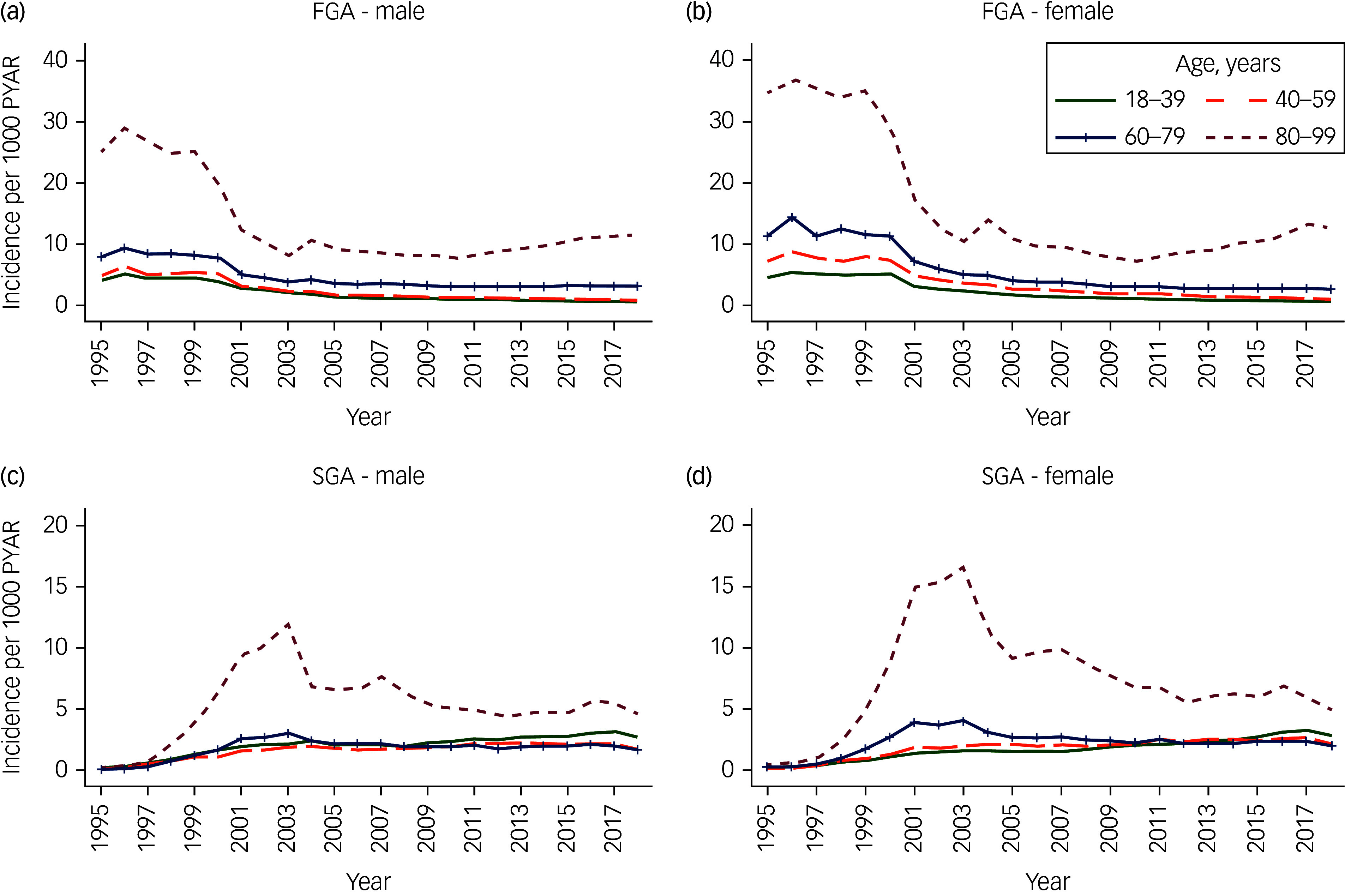
Unadjusted initiation of first-generation antipsychotic (FGA) (a, b) and second-generation antipsychotic (SGA) (c, d) drug prescribing per 1000 person-years at risk (PYAR), from 1995 to 2018, by age groups (years), stratified by gender.

**Fig. 3:**
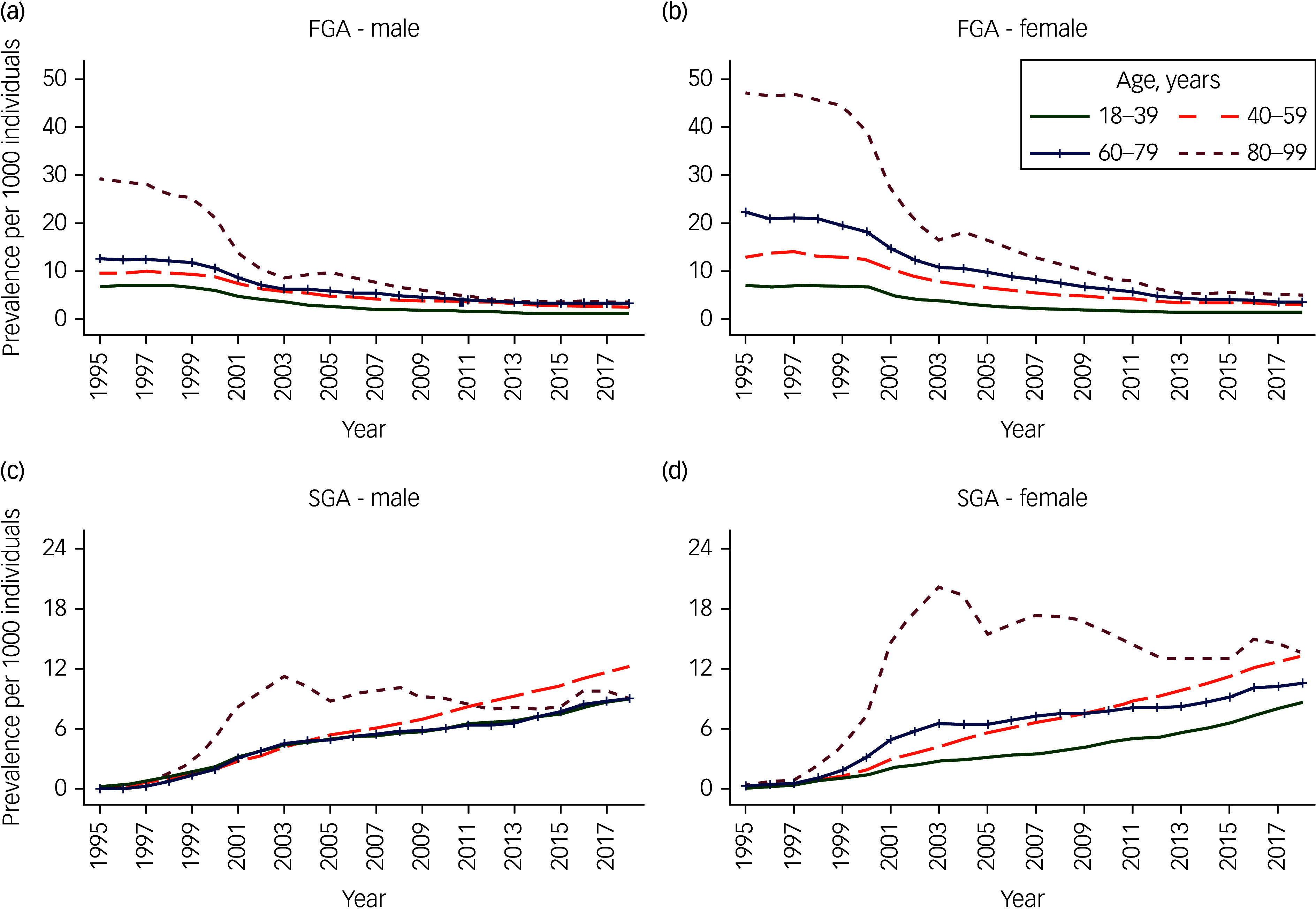
Unadjusted prevalence of first-generation antipsychotic (FGA) (a, b) and second-generation antipsychotic (SGA) (c, d) drug prescribing per 1000 individuals, from 1995 to 2018, by age groups (years), stratified by gender.

As we found the interactions to be significant, all analyses were stratified by gender (Supplementary Appendix 3). Figures 1–3 in our study described the unadjusted initiation and prevalence of antipsychotic drug prescribing stratified by gender, over the calendar years; further subgrouped into subclasses in Fig. 1, and into age bands in Figs. 2 and 3. More detailed information on antipsychotic drug prescribing initiation and prevalence can be found in Supplementary Appendices 4–8. All analyses were carried out with Stata software, version 17.0 for Windows and macOS.

### Ethics

Approval for using IMRD was granted by the NHS Health Research Authority (NHS Research Ethics Committee reference: 18/LO/0441). Science approval to undertake this study was obtained from the IMRD’s Scientific Review Committee (SRC) in February 2022 (SRC reference number: 22SRC009). All data utilised in this study were anonymised and no individual consent was needed.

## Results

### Demographics

There were 408 184 and 1 927 626 eligible individuals included in the study in 1995 and 2018, respectively (Supplementary Appendix 2). Of these, just over 50% were women (Supplementary Appendix 2). In 1995, about 71.2% of the individuals were aged between 18 and 59 years, and 28.8% were aged between 60 and 99 years. In 2018, 67.5 and 32.5% of the individuals were between the ages of 18–59 years and 60–99 years, respectively (Supplementary Appendix 2).

### Initiation

In the late 1990s, antipsychotic drug initiation remained stable at 6–7/1000 PYAR (95% CI 5.73–7.86) for men, and at 9–11/1000 PYAR (95% CI 8.87–11.47) in women (Supplementary Appendix 4a). From 2001, the initiation of antipsychotic treatment decreased gradually, but became stable from 2005 at 4/1000 PYAR (95% CI 3.77–4.53) in men and 4–5/1000 PYAR (95% CI 4.29–5.40) in women (Supplementary Appendix 4a). Throughout the study period, antipsychotic drug initiation was greater among women compared with men, but increased with age in both genders: male individuals aged 80–99 years were five times more likely (IRR 4.85, 95% CI 4.75–4.95) to be initiated antipsychotic drugs compared with male individuals aged 18–39 years; and female individuals aged 80–99 years were six times more likely (IRR 5.90, 95% CI 5.78–6.02) compared with female individuals aged 18–39 years (Supplementary Appendix 4a). Individuals from the most deprived backgrounds (Townsend score of 5) were more than twice as likely to be initiated antipsychotic drugs across both men (IRR 2.69, 95% CI 2.62–2.75) and women (IRR 2.19, 95% CI 2.15–2.24).

### By subclasses

The initiation of FGAs and SGAs showed contrasting trends over the study period across both men and women (Fig. 1(a) and (b)). The initiation of FGAs peaked in 1996 at 7.44/1000 PYAR (95% CI 7.13–7.76) in men and 11.04/1000 PYAR (95% CI 10.68–11.42) in women, before a sharp fall between 2000 and 2004 (Supplementary Appendix 5a). FGAs remained relatively stable thereafter at around 2/1000 PYAR (95% CI 1.84–2.50) in men and 2–3/1000 PYAR (95% CI 2.17–3.46) in women (Supplementary Appendix 5a). Conversely, between 1995 and 2003, the initiation of SGAs increased by just over 15 times (IRR 14.58, 95% CI 10.72–19.83) in men and 19 times (IRR 19.47, 95% CI 14.18–26.74) in women (Supplementary Appendix 6a). It then remained consistent at 2–3/1000 PYAR in both men (95% CI 2.04–2.79) and women (95% CI 2.46–3.18). Individuals across both genders were two times more likely to be initiated FGAs, and two to three times more likely to be initiated SGAs, if they were from the most deprived backgrounds (Townsend score of 5) compared with those from the least deprived backgrounds (Townsend score 1) (Supplementary Appendices 5a and 6a).

Overall, the initiation of both FGAs and SGAs was much greater in individuals aged 80–99 years compared with those aged 18–39 years (Fig. 2): by more than seven-fold in men (IRR 7.78, 95% CI 7.56–8.00) and women (IRR 7.45, 95% CI 7.25–7.66) for FGAs (Supplementary Appendix 5a), and by three-fold (IRR 2.77, 95% CI 2.69–2.85) in men and four-fold (IRR 4.43, 95% CI 4.32–4.54) in women for SGAs (Supplementary Appendix 6a). However, SGA initiation in the oldest age group showed a sharp decline between 2003 and 2005, followed by a less sharp decline up to 2018 (Fig. 2(c) and (d)).

### Prevalence

Overall, the prevalence of antipsychotic prescribing has remained relatively consistent throughout the study period (Fig. 1(c) and (d), Supplementary Appendix 4b). Although in the first decade of the 2000s, there was a slight decline in antipsychotic drug prevalence among women, but by 2018, the prevalence for both men and women was close to that of the late 1990s, at around 12/1000 for men (95% CI 11.93–12.38) and 14/1000 for women (95% CI 13.28–13.74) (Supplementary Appendix 4b).

Similar to their initiation, antipsychotic drug prevalence was greater in women compared with men, but increased with age in both gender: male individuals aged 80–99 years were two times more likely (PRR 2.22, 95% CI 2.19–2.25) to be prescribed antipsychotic drugs compared with male individuals aged 18–39 years, and female individuals aged 80–99 years were four times more likely (PRR 4.28, 95% CI 4.24–4.33) compared with females aged 18–39 years (Supplementary Appendix 4b). Individuals from the most deprived backgrounds (Townsend score of 5) were four-fold more likely (PRR 3.87, 95% CI 3.82–3.92) to be prescribed antipsychotic drugs if they were men, and three-fold more likely (PRR 2.80, 95% CI 2.77–2.83) if they were women, compared with those from the least deprived backgrounds (Townsend score of 1) (Supplementary Appendix 4b).

### By subclasses

The prevalence of SGAs increased throughout the study period and overtook the prevalence of FGAs by 2005/2006 (Fig. 1(c) and (d)). By 2018, SGAs were prescribed at around 10/1000 individuals (95% CI 10.02–10.43) in men, and 11/1000 individuals (95% CI 11.00–11.41) in women (Supplementary Appendix 6b). Simultaneously, FGAs were prescribed at around 2/1000 individuals (95% CI 2.26–2.46) in men, and 3/1000 individuals (95% CI 2.71–2.92) in women (Supplementary Appendix 5b). Overall, the prevalence of both FGAs and SGAs was higher in individuals aged 80–99 years compared with those aged 18–39 years (Fig. 3): by three-fold among men (PRR 3.42, 95% CI 3.35–3.50) and five-fold among women (PRR 5.36, 95% CI 5.27–5.45) for FGAs (Supplementary Appendix 5b), and by nearly two times among men (PRR 1.67, 95% CI 1.64–1.70) and nearly four times among women (PRR 3.56, 95% CI 3.51–3.61) for SGAs (Supplementary Appendix 6b). However, the prevalence of SGAs declined rapidly between 2003 and 2005 among individuals aged 80–99 years (Fig. 3(c) and (d)). After 2011, the prevalence of SGAs among male individuals aged 40–59 years exceeded all other age groups (Fig. 3(c)). Individuals were three to four times more likely to be initiated both FGAs and SGAs if they were from the most deprived backgrounds (Townsend score of 5) compared with those from the least deprived backgrounds (Townsend score of 1) (Supplementary Appendices 5b and 6b).

## Discussion

### Summary of key findings

Initiation of antipsychotic drug prescribing was relatively stable for both men and women in the late 1990s. From 2001, the initiation declined and remained stable again from 2005, at around 4/1000 PYAR in men and 4–5/1000 PYAR in women. Conversely, the prevalence of antipsychotic drug prescribing remained relatively constant throughout the study period, reaching 12/1000 in men and 14/1000 in women by 2018. FGA prescribing dominated the overall initiation and prevalence across both men and women up to the early 2000s, at which point SGAs became dominant. The initiation and prevalence of antipsychotic drug prescribing was higher among women compared to men, but increased with age in both genders. Individuals from more deprived backgrounds were more likely to be initiated and prescribed antipsychotic medication.

### Comparison with existing literature

Our findings are consistent to the results from previous studies.^4–6,8–10^ A previous study looking at the UK population found consistent prevalence at approximately 1% between 2007 and 2011.^4^ These trends in our study may have been influenced by the rapid reduction in the initiation of FGAs between 2000 and 2004, coupled with a substantial rise in the initiation of SGAs. Similar trends were reported in another UK study, where the rate of SGA prescribing increased by tenfold between 1993 and 1999; however, they only included individuals with schizophrenia or schizoaffective disorder diagnosis.^8^ Increased SGA initiation was in line with the inception of SGA approval for the use in the treatment of schizophrenia over FGAs (because of their safer side-effects profile) in the early 2000s,^11^ and the recommendation made by the NICE in 2002.^22^ Despite the updated treatment recommendation in the NICE guideline (CG82) in 2009,^23^ which was that any antipsychotic drug can be prescribed for newly diagnosed schizophrenia, the initiation and prevalence of FGA prescribing did not seem to be affected by these changes. The initiation of FGAs and SGAs were equal by 2003, and this pattern continued to the end of the study. However, prevalence of SGAs increased rapidly whereas prevalence of FGAs declined. Thus, SGAs overtook the prevalence of FGAs between 2003 and 2006. Overall, this may suggest that SGAs were prescribed for a longer duration compared with FGAs.

The decline in SGA prevalence among older individuals after 2003 may be a result of the safety warnings released in the early 2000s. Specific warnings were issued on the exposure of risperidone and olanzapine, and increased risk of stroke among individuals with dementia.^12,13,24^ However, findings from subsequent reviews suggested that antipsychotic drug use increases risk of cerebrovascular accident among the elderly population, irrespective of dementia diagnosis in general.^15,25^ In terms of the safety profiles of FGAs versus SGAs, the previous reports showed mixed findings.^25,26^ This is an area that still requires further research.

Our findings reflect the safety warnings issued in 2004.^14^ Thus, the initiation and prevalence of SGAs declined rapidly between 2003 and 2005 among the elderly population. However, overall initiation and prevalence of SGA prescribing remained much higher in the female elderly population (80–99 years). This is not surprising, as the prevalence of all-type dementia and Alzheimer’s disease are greater in women compared with men.^15^ Other studies also found that antipsychotic drugs continued to be prescribed among the older population and in those with a diagnosis of dementia.^4^ It is worth noting that risperidone, an SGA, remains the only antipsychotic approved as the therapeutic choice for the management of behavioural and psychological symptoms of dementia in the UK.^27^ Therefore, clinical judgement using risk-benefit is crucial to ensure this group of individuals will receive the effective, yet safest treatment.

### Strengths and limitations

The strength of this study is that it represents real-life prescribing of antipsychotic medication in primary care in the UK. In some cases, it is likely that antipsychotic medication is initiated in secondary care, which will not be captured in our data. However, the prescribing budget lies with the primary care, and thus continuation of treatment will be recorded in primary care data. We would also be missing data on prescriptions of clozapine and long-acting injectables, which are typically given in secondary care only. Our study did not examine the indication for the treatment. As such, all prescriptions, including those for off-label purposes, will be included in our analyses, and we cannot assess whether the prescribing of the antipsychotics was appropriate. Initiation estimates may be slightly underestimated because we sourced the data from some practices coming from more affluent areas.^18^ However, we adjusted for socioeconomic deprivation in our relative estimates, to reduce this bias.

### Implications for research and practice

We encourage further research from data sources where it is possible to link prescribing records of secondary care to those of primary care, to ensure all antipsychotics, including clozapine and long-acting injections (depot), are included. Further research should focus on the prescribing of antipsychotics in the older female population and the safety thereof. We also need more detailed understanding of the prescribing of individual antipsychotic medication including dosage. And finally, we would encourage further research on the length of treatment at an individual level. Antipsychotic drug initiation and prevalence may vary internationally because of differences in local prescribing guidelines. Nonetheless, our findings may inform prescribing practices globally. Notably, NICE guidelines are frequently referenced by other countries when developing their own prescribing policies.

## Supporting information

Samsuddin et al. supplementary material Samsuddin et al. supplementary material

## Data Availability

The data that support the findings of this study are not publicly available or available on request due to privacy or ethical restrictions, but can be requested from the IQVIA Medical Research Data. Access to the data requires a protocol and is subject to ethical approval.

## Supplementary material

The supplementary material is available online at https://doi.org/10.1192/bjo.2025.10815
