# Supplementary material for: An overview of antipsychotic drug prescribing trends (initiation/prevalence) in UK primary care from 1995 to 2018: analysis of electronic health records from over 790 general practices
Source: BJPsych Open. 2025 Aug 14;11(5):e180. doi: 10.1192/bjo.2025.10815 (PMC12451550; doi:10.1192/bjo.2025.10815)

**Manuscript title**

An overview of antipsychotic prescribing trends (initiation/prevalence) in UK primary care from 1995 to 2018: An analysis of electronic health records from over 790 general practices.

**Appendix 1.** The list of antipsychotic medication examined.

| Sub-class | Medication name | |
| --- | --- | --- |
| Typical (First-Generation) | Benperidol  Chlorpromazine  Chlorprothixene  Droperidol  Flupentixol  Fluphenazine  Fluspirilene  Haloperidol  Levomepromazine  Loxapine  Oxypertine  Pericyazine | Perphenazine  Pimozide  Pipotiazine  Promazine  Sulpiride  Thiopropazate  Thioproperazine  Thioridazine  Trifluoperazine  Trifluperidol  Zotepin  Zuclopentixol |
| Atypical (Second-Generation) | Amisulpride  Aripiprazole  Asenapine  Clozapine  Lurasidone  Olanzapine | Paliperidone  Quetiapine  Remoxipride  Risperidone  Sertindole |

**Appendix 2.** Demographics of the participants in year 1995 and 2018.

| N | Year | | | |
| --- | --- | --- | --- | --- |
|  | 1995 | | 2018 | |
|  | 408,184 | | 1,927,626 | |
| Sex (%) |  |  |  |  |
| Male | 197,129 | (48.3) | 929,357 | (48.2) |
| Female | 211,055 | (51.7) | 998,269 | (51.8) |
| Townsend score (%) |  |  |  |  |
| 1 (least deprived) | 117,011 | (28.7) | 413,242 | (21.4) |
| 2 | 91,038 | (22.3) | 422,954 | (21.9) |
| 3 | 78,746 | (19.3) | 443,569 | (23.0) |
| 4 | 69,903 | (17.1) | 373,129 | (19.4) |
| 5 (most deprived) | 51,486 | (12.6) | 274,732 | (14.3) |
| Age band (%) |  |  |  |  |
| 18-39 | 149,951 | (36.7) | 625,136 | (32.4) |
| 40-59 | 140,712 | (34.5) | 676,343 | (35.1) |
| 60-79 | 93,936 | (23.0) | 479,791 | (24.9) |
| 80-99 | 23,585 | (5.8) | 146,356 | (7.6) |

**Appendix 3.** Log likelihood ratio tests to examine interactions between sex and age, year, and socioeconomic deprivation (Townsend score)

**Antipsychotic initiation:**

1. Interaction between sex and age

*lrtest m1a m1b*

*Likelihood-ratio test*

*LR chi2(3) = 873.71*

*Prob > chi2 = 0.0000*

1. Interaction between sex and year

*lrtest m1c m1d*

*Likelihood-ratio test*

*LR chi2(23) = 868.48*

*Prob > chi2 = 0.0000*

1. Interaction between sex and socioeconomic deprivation (Townsend score)

*lrtest m1e m1f*

*Likelihood-ratio test*

*LR chi2(4) = 321.62*

*Prob > chi2 = 0.0000*

**Antipsychotic prevalence:**

1. Interaction between sex and age

*lrtest m1a m1b*

*Likelihood-ratio test*

*LR chi2(3) = 7888.64*

*Prob > chi2 = 0.0000*

1. Interaction between sex and year

*lrtest m1c m1d*

*Likelihood-ratio test*

*LR chi2(23) = 1760.09*

*Prob > chi2 = 0.0000*

1. Interaction between sex and socioeconomic deprivation (Townsend score)

*lrtest m1e m1f*

*Likelihood-ratio test*

*LR chi2(4) = 2293.77*

*Prob > chi2 = 0.0000*

**Appendix 4.**

(a) Unadjusted initiation of antipsychotic prescribing per 1000 person-years at risk (PYAR) and adjusted Incidence Rate Ratio (IRR), stratified by sex.

| (Year) | (a) Initiation | | | | | | | |
| --- | --- | --- | --- | --- | --- | --- | --- | --- |
|  | Male | | | | Female | | | |
|  | I | CI (95%) | aIRR | CI (95%) | I | CI (95%) | aIRR | CI (95%) |
| 1995 | 6.05 | [5.73, 6.37] | 1 |  | 9.25 | [8.87, 9.64] | 1 |  |
| 1996 | 7.54 | [7.23, 7.86] | 1.23 | [1.13,1.35] | 11.10 | [10.73, 11.47] | 1.18 | [1.09,1.29] |
| 1997 | 6.54 | [6.29, 6.80] | 1.07 | [0.98,1.16] | 10.01 | [9.70, 10.32] | 1.07 | [0.98,1.15] |
| 1998 | 6.78 | [6.55, 7.02] | 1.11 | [1.02,1.21] | 10.07 | [9.79, 10.35] | 1.08 | [1.00,1.17] |
| 1999 | 7.09 | [6.88, 7.30] | 1.17 | [1.08,1.26] | 10.53 | [10.29, 10.78] | 1.14 | [1.06,1.22] |
| 2000 | 6.78 | [6.61, 6.96] | 1.11 | [1.03,1.20] | 10.24 | [10.04, 10.45] | 1.11 | [1.03,1.19] |
| 2001 | 5.17 | [5.03, 5.31] | 0.85 | [0.78,0.91] | 7.67 | [7.51, 7.83] | 0.82 | [0.77,0.88] |
| 2002 | 5.09 | [4.97, 5.22] | 0.83 | [0.77,0.89] | 7.01 | [6.87, 7.15] | 0.75 | [0.70,0.80] |
| 2003 | 4.87 | [4.75, 4.98] | 0.79 | [0.73,0.85] | 6.67 | [6.54, 6.81] | 0.71 | [0.66,0.76] |
| 2004 | 4.62 | [4.52, 4.73] | 0.74 | [0.69,0.80] | 6.01 | [5.89, 6.13] | 0.64 | [0.60,0.68] |
| 2005 | 4.08 | [3.99, 4.18] | 0.65 | [0.61,0.70] | 5.29 | [5.18, 5.40] | 0.56 | [0.52,0.60] |
| 2006 | 3.98 | [3.89, 4.07] | 0.63 | [0.59,0.68] | 5.02 | [4.92, 5.12] | 0.53 | [0.49,0.57] |
| 2007 | 4.10 | [4.00, 4.19] | 0.65 | [0.60,0.70] | 5.09 | [4.99, 5.19] | 0.53 | [0.50,0.57] |
| 2008 | 3.87 | [3.78, 3.96] | 0.61 | [0.56,0.66] | 4.75 | [4.65, 4.85] | 0.50 | [0.46,0.53] |
| 2009 | 3.93 | [3.84, 4.02] | 0.61 | [0.57,0.66] | 4.65 | [4.55, 4.74] | 0.48 | [0.45,0.52] |
| 2010 | 3.86 | [3.77, 3.95] | 0.60 | [0.56,0.65] | 4.48 | [4.39, 4.57] | 0.46 | [0.43,0.50] |
| 2011 | 4.14 | [4.05, 4.23] | 0.64 | [0.59,0.69] | 4.75 | [4.66, 4.85] | 0.49 | [0.46,0.52] |
| 2012 | 3.89 | [3.80, 3.98] | 0.59 | [0.55,0.64] | 4.38 | [4.29, 4.47] | 0.45 | [0.42,0.48] |
| 2013 | 4.10 | [4.01, 4.20] | 0.62 | [0.58,0.67] | 4.59 | [4.50, 4.69] | 0.47 | [0.44,0.50] |
| 2014 | 4.13 | [4.03, 4.22] | 0.62 | [0.58,0.67] | 4.71 | [4.61, 4.81] | 0.48 | [0.45,0.51] |
| 2015 | 4.25 | [4.14, 4.35] | 0.63 | [0.59,0.68] | 4.82 | [4.72, 4.93] | 0.49 | [0.45,0.52] |
| 2016 | 4.41 | [4.29, 4.53] | 0.66 | [0.61,0.71] | 5.01 | [4.89, 5.13] | 0.51 | [0.47,0.55] |
| 2017 | 4.26 | [4.14, 4.38] | 0.64 | [0.59,0.69] | 5.03 | [4.91, 5.16] | 0.52 | [0.48,0.55] |
| 2018 | 3.97 | [3.84, 4.09] | 0.59 | [0.54,0.64] | 4.60 | [4.47, 4.73] | 0.47 | [0.43,0.50] |
| (Age band) |  |  |  |  |  |  |  |  |
| 18-39 | 3.42 | [3.39, 3.45] | 1 |  | 3.30 | [3.27, 3.33] | 1 |  |
| 40-59 | 3.35 | [3.31, 3.38] | 1.05 | [1.03,1.07] | 4.25 | [4.21, 4.28] | 1.37 | [1.35,1.40] |
| 60-79 | 5.45 | [5.40, 5.51] | 1.74 | [1.71,1.77] | 6.26 | [6.21, 6.32] | 2.02 | [1.98,2.05] |
| 80-99 | 14.95 | [14.76, 15.14] | 4.85 | [4.75,4.95] | 18.35 | [18.20, 18.51] | 5.90 | [5.78,6.02] |
| (Townsend score) |  |  |  |  |  |  |  |  |
| 1 | 3.06 | [3.02, 3.10] | 1 |  | 4.16 | [4.11, 4.20] | 1 |  |
| 2 | 3.69 | [3.64, 3.73] | 1.20 | [1.17,1.22] | 4.82 | [4.77, 4.87] | 1.14 | [1.12,1.16] |
| 3 | 4.26 | [4.21, 4.31] | 1.44 | [1.41,1.47] | 5.58 | [5.53, 5.64] | 1.37 | [1.34,1.39] |
| 4 | 5.43 | [5.37, 5.49] | 1.86 | [1.82,1.90] | 6.71 | [6.64, 6.77] | 1.65 | [1.61,1.68] |
| 5 | 7.56 | [7.48, 7.65] | 2.69 | [2.64,2.75] | 8.56 | [8.47, 8.65] | 2.19 | [2.15,2.24] |

I – Initiation; aIRR – Adjusted Incidence Rate Ratio.

All rates were adjusted for other characteristics in this table.

(b) Unadjusted prevalence of antipsychotic prescribing per 1000 individuals and adjusted Prevalence Rate Ratio (PRR), stratified by sex.

| (Year) | (b) Prevalence | | | | | | | |
| --- | --- | --- | --- | --- | --- | --- | --- | --- |
|  | Male | | | | Female | | | |
|  | P | CI (95%) | aPRR | CI (95%) | P | CI (95%) | aPRR | CI (95%) |
| 1995 | 10.08 | [9.65, 10.54] | 1 |  | 15.79 | [15.26, 16.34] | 1 |  |
| 1996 | 10.21 | [9.81, 10.63] | 1.02 | [0.96,1.09] | 15.72 | [15.23, 16.22] | 1.00 | [0.96,1.05] |
| 1997 | 10.61 | [10.25, 10.97] | 1.05 | [0.99,1.11] | 16.36 | [15.93, 16.79] | 1.03 | [0.98,1.07] |
| 1998 | 10.54 | [10.22, 10.87] | 1.05 | [1.00,1.11] | 16.08 | [15.70, 16.47] | 1.02 | [0.98,1.06] |
| 1999 | 10.66 | [10.37, 10.96] | 1.06 | [1.01,1.12] | 16.09 | [15.75, 16.44] | 1.02 | [0.98,1.06] |
| 2000 | 10.23 | [9.99, 10.47] | 1.03 | [0.98,1.08] | 15.60 | [15.31, 15.89] | 1.00 | [0.96,1.04] |
| 2001 | 9.27 | [9.07, 9.47] | 0.93 | [0.88,0.97] | 13.87 | [13.64, 14.11] | 0.88 | [0.85,0.92] |
| 2002 | 9.00 | [8.83, 9.18] | 0.90 | [0.86,0.94] | 12.91 | [12.71, 13.12] | 0.82 | [0.79,0.85] |
| 2003 | 9.11 | [8.95, 9.28] | 0.91 | [0.86,0.95] | 12.65 | [12.46, 12.84] | 0.80 | [0.77,0.84] |
| 2004 | 9.24 | [9.08, 9.40] | 0.91 | [0.87,0.96] | 12.42 | [12.24, 12.60] | 0.79 | [0.76,0.82] |
| 2005 | 9.14 | [8.99, 9.28] | 0.90 | [0.85,0.94] | 11.95 | [11.78, 12.11] | 0.75 | [0.72,0.78] |
| 2006 | 9.25 | [9.11, 9.40] | 0.90 | [0.86,0.95] | 11.84 | [11.68, 12.00] | 0.74 | [0.71,0.77] |
| 2007 | 9.24 | [9.10, 9.38] | 0.90 | [0.86,0.94] | 11.77 | [11.62, 11.93] | 0.73 | [0.71,0.76] |
| 2008 | 9.32 | [9.18, 9.46] | 0.90 | [0.86,0.95] | 11.61 | [11.46, 11.76] | 0.72 | [0.70,0.75] |
| 2009 | 9.42 | [9.28, 9.56] | 0.91 | [0.87,0.96] | 11.54 | [11.39, 11.69] | 0.72 | [0.69,0.74] |
| 2010 | 9.62 | [9.48, 9.76] | 0.92 | [0.88,0.97] | 11.50 | [11.35, 11.65] | 0.71 | [0.68,0.74] |
| 2011 | 9.80 | [9.66, 9.94] | 0.94 | [0.89,0.98] | 11.47 | [11.32, 11.62] | 0.71 | [0.68,0.73] |
| 2012 | 9.83 | [9.68, 9.97] | 0.93 | [0.89,0.97] | 11.12 | [10.97, 11.26] | 0.68 | [0.65,0.71] |
| 2013 | 10.04 | [9.89, 10.19] | 0.95 | [0.90,0.99] | 11.22 | [11.07, 11.37] | 0.68 | [0.66,0.71] |
| 2014 | 10.29 | [10.13, 10.44] | 0.97 | [0.92,1.01] | 11.55 | [11.40, 11.71] | 0.70 | [0.68,0.73] |
| 2015 | 10.70 | [10.52, 10.87] | 0.99 | [0.94,1.04] | 12.14 | [11.97, 12.32] | 0.73 | [0.70,0.76] |
| 2016 | 11.47 | [11.28, 11.67] | 1.06 | [1.01,1.11] | 12.94 | [12.74, 13.14] | 0.78 | [0.75,0.81] |
| 2017 | 11.74 | [11.53, 11.95] | 1.08 | [1.03,1.13] | 13.11 | [12.90, 13.33] | 0.79 | [0.76,0.82] |
| 2018 | 12.15 | [11.93, 12.38] | 1.11 | [1.05,1.16] | 13.51 | [13.28, 13.74] | 0.81 | [0.77,0.84] |
| (Age band) |  |  |  |  |  |  |  |  |
| 18-39 | 7.66 | [7.60, 7.71] | 1 |  | 6.54 | [6.49, 6.59] | 1 |  |
| 40-59 | 10.79 | [10.73, 10.85] | 1.52 | [1.51,1.54] | 12.49 | [12.42, 12.56] | 2.06 | [2.04,2.08] |
| 60-79 | 10.51 | [10.43, 10.59] | 1.53 | [1.51,1.55] | 14.71 | [14.63, 14.80] | 2.43 | [2.40,2.45] |
| 80-99 | 15.25 | [15.06, 15.45] | 2.22 | [2.19,2.25] | 26.36 | [26.17, 26.55] | 4.28 | [4.24,4.33] |
| (Townsend score) |  |  |  |  |  |  |  |  |
| 1 | 5.49 | [5.44, 5.54] | 1 |  | 8.32 | [8.26, 8.38] | 1 |  |
| 2 | 7.16 | [7.09, 7.22] | 1.30 | [1.29,1.32] | 9.69 | [9.62, 9.77] | 1.16 | [1.14,1.17] |
| 3 | 9.33 | [9.25, 9.41] | 1.73 | [1.71,1.76] | 12.00 | [11.91, 12.08] | 1.48 | [1.47,1.50] |
| 4 | 12.97 | [12.87, 13.07] | 2.44 | [2.41,2.47] | 15.59 | [15.49, 15.69] | 1.96 | [1.94,1.98] |
| 5 | 20.20 | [20.06, 20.35] | 3.87 | [3.82,3.92] | 21.38 | [21.24, 21.53] | 2.80 | [2.77,2.83] |

P – Prevalence; aPRR – Adjusted Prevalence Rate Ratio.

All rates were adjusted for other characteristics in this table.

**Appendix 5.**

1. Unadjusted initiation of First-Generation Antipsychotic (FGA) per 1000 person-years at risk (PYAR) and adjusted Incidence Rate Ratio (IRR) stratified by sex.

|  | (a) Initiation | | | | | | | |
| --- | --- | --- | --- | --- | --- | --- | --- | --- |
|  | Male | | | | Female | | | |
| (Year) | I | CI (95%) | aIRR | CI (95%) | I | CI (95%) | aIRR | CI (95%) |
| 1995 | 6.01 | [5.69, 6.33] | 1 |  | 9.20 | [8.83, 9.59] | 1 |  |
| 1996 | 7.44 | [7.13, 7.76] | 1.22 | [1.12,1.34] | 11.04 | [10.68, 11.42] | 1.18 | [1.09,1.29] |
| 1997 | 6.37 | [6.12, 6.63] | 1.05 | [0.96,1.14] | 9.83 | [9.53, 10.14] | 1.05 | [0.97,1.14] |
| 1998 | 6.41 | [6.18, 6.64] | 1.05 | [0.96,1.15] | 9.69 | [9.42, 9.97] | 1.04 | [0.96,1.13] |
| 1999 | 6.40 | [6.21, 6.61] | 1.06 | [0.97,1.15] | 9.85 | [9.61, 10.09] | 1.07 | [0.99,1.15] |
| 2000 | 5.89 | [5.73, 6.05] | 0.97 | [0.90,1.05] | 9.08 | [8.88, 9.27] | 0.99 | [0.92,1.06] |
| 2001 | 3.89 | [3.77, 4.01] | 0.64 | [0.59,0.69] | 5.86 | [5.72, 6.00] | 0.63 | [0.59,0.68] |
| 2002 | 3.40 | [3.30, 3.50] | 0.55 | [0.51,0.60] | 4.71 | [4.60, 4.83] | 0.51 | [0.47,0.54] |
| 2003 | 2.90 | [2.81, 2.99] | 0.47 | [0.43,0.51] | 4.05 | [3.94, 4.15] | 0.43 | [0.40,0.46] |
| 2004 | 2.87 | [2.79, 2.96] | 0.46 | [0.43,0.50] | 4.11 | [4.01, 4.21] | 0.44 | [0.41,0.47] |
| 2005 | 2.42 | [2.35, 2.50] | 0.39 | [0.36,0.42] | 3.38 | [3.29, 3.46] | 0.36 | [0.33,0.38] |
| 2006 | 2.29 | [2.22, 2.36] | 0.36 | [0.33,0.39] | 3.06 | [2.98, 3.14] | 0.32 | [0.30,0.35] |
| 2007 | 2.26 | [2.19, 2.33] | 0.35 | [0.33,0.38] | 2.98 | [2.90, 3.05] | 0.31 | [0.29,0.34] |
| 2008 | 2.16 | [2.09, 2.22] | 0.34 | [0.31,0.36] | 2.71 | [2.64, 2.78] | 0.28 | [0.26,0.30] |
| 2009 | 2.14 | [2.07, 2.20] | 0.33 | [0.31,0.36] | 2.54 | [2.47, 2.61] | 0.26 | [0.25,0.28] |
| 2010 | 1.98 | [1.91, 2.04] | 0.30 | [0.28,0.33] | 2.34 | [2.27, 2.41] | 0.24 | [0.22,0.26] |
| 2011 | 2.09 | [2.03, 2.15] | 0.32 | [0.29,0.34] | 2.47 | [2.41, 2.54] | 0.25 | [0.24,0.27] |
| 2012 | 1.93 | [1.87, 1.99] | 0.29 | [0.27,0.31] | 2.31 | [2.25, 2.38] | 0.24 | [0.22,0.26] |
| 2013 | 1.95 | [1.89, 2.01] | 0.29 | [0.27,0.32] | 2.24 | [2.17, 2.30] | 0.23 | [0.21,0.25] |
| 2014 | 1.94 | [1.87, 2.00] | 0.29 | [0.26,0.31] | 2.33 | [2.26, 2.40] | 0.24 | [0.22,0.26] |
| 2015 | 2.04 | [1.97, 2.11] | 0.30 | [0.27,0.32] | 2.40 | [2.33, 2.48] | 0.24 | [0.22,0.26] |
| 2016 | 2.03 | [1.96, 2.11] | 0.30 | [0.28,0.33] | 2.35 | [2.27, 2.43] | 0.24 | [0.22,0.26] |
| 2017 | 1.92 | [1.84, 2.01] | 0.28 | [0.26,0.31] | 2.40 | [2.31, 2.49] | 0.25 | [0.23,0.27] |
| 2018 | 1.95 | [1.87, 2.04] | 0.28 | [0.26,0.31] | 2.35 | [2.26, 2.45] | 0.24 | [0.22,0.26] |
| (Age band) |  |  |  |  |  |  |  |  |
| 18-39 | 1.52 | [1.50, 1.54] | 1 |  | 1.73 | [1.70, 1.75] | 1 |  |
| 40-59 | 1.91 | [1.88, 1.93] | 1.35 | [1.31,1.39] | 2.65 | [2.62, 2.68] | 1.65 | [1.60,1.70] |
| 60-79 | 3.88 | [3.84, 3.93] | 2.80 | [2.73,2.87] | 4.35 | [4.31, 4.40] | 2.68 | [2.61,2.75] |
| 80-99 | 10.33 | [10.18, 10.49] | 7.78 | [7.56,8.00] | 11.94 | [11.81, 12.06] | 7.45 | [7.25,7.66] |
| (Townsend score) |  |  |  |  |  |  |  |  |
| 1 | 1.95 | [1.92, 1.98] | 1 |  | 2.72 | [2.68, 2.75] | 1 |  |
| 2 | 2.34 | [2.30, 2.38] | 1.21 | [1.17,1.24] | 3.13 | [3.09, 3.17] | 1.14 | [1.11,1.17] |
| 3 | 2.54 | [2.51, 2.58] | 1.40 | [1.36,1.44] | 3.49 | [3.45, 3.53] | 1.33 | [1.30,1.36] |
| 4 | 3.11 | [3.07, 3.16] | 1.75 | [1.71,1.80] | 4.13 | [4.08, 4.18] | 1.58 | [1.54,1.63] |
| 5 | 4.15 | [4.09, 4.22] | 2.46 | [2.39,2.54] | 5.23 | [5.16, 5.30] | 2.10 | [2.04,2.16] |

I – Initiation; aIRR – Adjusted Incidence Rate Ratio.

All rates were adjusted for other characteristics in this table.

1. Unadjusted prevalence of First-Generation Antipsychotic prescribing (FGA) per 1000 individuals and adjusted Prevalence Rate Ratio (PRR) stratified by sex.

|  | (b) Prevalence | | | | | | | |
| --- | --- | --- | --- | --- | --- | --- | --- | --- |
|  | Male | | | | Female | | | |
| (Year) | P | CI (95%) | aPRR | CI (95%) | P | CI (95%) | aPRR | CI (95%) |
| 1995 | 10.00 | [9.56, 10.45] | 1 |  | 15.73 | [15.20, 16.27] | 1 |  |
| 1996 | 10.07 | [9.67, 10.49] | 1.02 | [0.96,1.08] | 15.61 | [15.12, 16.10] | 1.00 | [0.95,1.05] |
| 1997 | 10.29 | [9.94, 10.65] | 1.02 | [0.97,1.08] | 16.10 | [15.68, 16.53] | 1.02 | [0.97,1.06] |
| 1998 | 9.96 | [9.65, 10.28] | 1.00 | [0.95,1.06] | 15.57 | [15.20, 15.95] | 0.99 | [0.95,1.03] |
| 1999 | 9.68 | [9.40, 9.96] | 0.97 | [0.92,1.02] | 15.10 | [14.77, 15.44] | 0.96 | [0.92,1.00] |
| 2000 | 8.75 | [8.53, 8.97] | 0.88 | [0.84,0.93] | 13.99 | [13.72, 14.26] | 0.90 | [0.86,0.93] |
| 2001 | 7.02 | [6.85, 7.19] | 0.71 | [0.67,0.74] | 11.03 | [10.82, 11.24] | 0.71 | [0.68,0.74] |
| 2002 | 5.93 | [5.79, 6.08] | 0.60 | [0.57,0.63] | 9.03 | [8.86, 9.21] | 0.58 | [0.56,0.60] |
| 2003 | 5.30 | [5.17, 5.42] | 0.53 | [0.50,0.56] | 7.91 | [7.76, 8.06] | 0.51 | [0.49,0.53] |
| 2004 | 5.02 | [4.90, 5.14] | 0.50 | [0.47,0.52] | 7.61 | [7.47, 7.75] | 0.48 | [0.47,0.50] |
| 2005 | 4.59 | [4.49, 4.70] | 0.45 | [0.43,0.48] | 6.94 | [6.82, 7.07] | 0.44 | [0.42,0.46] |
| 2006 | 4.26 | [4.16, 4.36] | 0.42 | [0.40,0.44] | 6.28 | [6.17, 6.40] | 0.40 | [0.38,0.41] |
| 2007 | 3.94 | [3.85, 4.04] | 0.38 | [0.37,0.40] | 5.78 | [5.67, 5.89] | 0.36 | [0.35,0.38] |
| 2008 | 3.71 | [3.62, 3.80] | 0.36 | [0.34,0.38] | 5.25 | [5.15, 5.35] | 0.33 | [0.32,0.34] |
| 2009 | 3.56 | [3.47, 3.64] | 0.34 | [0.33,0.36] | 4.87 | [4.77, 4.96] | 0.30 | [0.29,0.32] |
| 2010 | 3.34 | [3.26, 3.42] | 0.32 | [0.30,0.34] | 4.47 | [4.38, 4.57] | 0.28 | [0.27,0.29] |
| 2011 | 3.17 | [3.09, 3.25] | 0.30 | [0.29,0.32] | 4.12 | [4.03, 4.21] | 0.25 | [0.24,0.27] |
| 2012 | 2.90 | [2.83, 2.98] | 0.27 | [0.26,0.29] | 3.63 | [3.55, 3.72] | 0.22 | [0.21,0.23] |
| 2013 | 2.72 | [2.65, 2.80] | 0.26 | [0.24,0.27] | 3.33 | [3.25, 3.41] | 0.20 | [0.20,0.21] |
| 2014 | 2.58 | [2.50, 2.65] | 0.24 | [0.23,0.25] | 3.18 | [3.09, 3.26] | 0.19 | [0.19,0.20] |
| 2015 | 2.51 | [2.43, 2.59] | 0.23 | [0.22,0.24] | 3.19 | [3.09, 3.28] | 0.19 | [0.18,0.20] |
| 2016 | 2.55 | [2.46, 2.64] | 0.23 | [0.22,0.25] | 3.13 | [3.04, 3.23] | 0.19 | [0.18,0.20] |
| 2017 | 2.33 | [2.24, 2.43] | 0.21 | [0.20,0.23] | 2.83 | [2.73, 2.93] | 0.17 | [0.16,0.18] |
| 2018 | 2.36 | [2.26, 2.46] | 0.21 | [0.20,0.23] | 2.82 | [2.71, 2.92] | 0.17 | [0.16,0.18] |
| (Age band) |  |  |  |  |  |  |  |  |
| 18-39 | 2.53 | [2.50, 2.56] | 1 |  | 2.62 | [2.59, 2.66] | 1 |  |
| 40-59 | 4.49 | [4.45, 4.53] | 1.95 | [1.92,1.98] | 5.76 | [5.72, 5.81] | 2.40 | [2.36,2.43] |
| 60-79 | 5.32 | [5.26, 5.37] | 2.39 | [2.35,2.43] | 8.08 | [8.02, 8.15] | 3.34 | [3.29,3.39] |
| 80-99 | 7.32 | [7.18, 7.45] | 3.42 | [3.35,3.50] | 13.00 | [12.87, 13.13] | 5.36 | [5.27,5.45] |
| (Townsend score) |  |  |  |  |  |  |  |  |
| 1 | 2.41 | [2.38, 2.45] | 1 |  | 4.19 | [4.15, 4.24] | 1 |  |
| 2 | 3.15 | [3.11, 3.19] | 1.34 | [1.31,1.37] | 4.82 | [4.77, 4.87] | 1.16 | [1.15,1.18] |
| 3 | 3.88 | [3.83, 3.93] | 1.74 | [1.71,1.78] | 5.67 | [5.62, 5.73] | 1.45 | [1.43,1.47] |
| 4 | 5.34 | [5.28, 5.40] | 2.46 | [2.41,2.50] | 7.47 | [7.40, 7.54] | 1.93 | [1.90,1.96] |
| 5 | 8.27 | [8.18, 8.37] | 3.93 | [3.86,4.01] | 9.93 | [9.83, 10.03] | 2.69 | [2.65,2.73] |

P – Prevalence; aPRR – Adjusted Prevalence Rate Ratio.

All rates were adjusted for other characteristics in this table.

**Appendix 6:**

1. Unadjusted initiation of Second-Generation Antipsychotic (SGA) prescribing per 1000 person-years at risk (PYAR) and adjusted Incidence Rate Ratio (IRR), stratified by sex.

|  | (a) Initiation | | | | | | | |
| --- | --- | --- | --- | --- | --- | --- | --- | --- |
|  | Male | | | | Female | | | |
| (Year) | I | CI (95%) | aIRR | CI (95%) | I | CI (95%) | aIRR | CI (95%) |
| 1995 | 0.18 | [0.13, 0.24] | 1 |  | 0.18 | [0.13, 0.24] | 1 |  |
| 1996 | 0.28 | [0.22, 0.34] | 1.54 | [1.06,2.25] | 0.25 | [0.19, 0.31] | 1.37 | [0.93,2.02] |
| 1997 | 0.50 | [0.44, 0.58] | 2.81 | [2.01,3.93] | 0.44 | [0.38, 0.51] | 2.44 | [1.72,3.46] |
| 1998 | 0.90 | [0.82, 0.99] | 5.05 | [3.66,6.96] | 0.87 | [0.79, 0.95] | 4.83 | [3.47,6.73] |
| 1999 | 1.28 | [1.19, 1.37] | 7.16 | [5.23,9.81] | 1.36 | [1.27, 1.45] | 7.62 | [5.51,10.54] |
| 2000 | 1.60 | [1.51, 1.68] | 8.94 | [6.55,12.19] | 2.08 | [1.99, 2.17] | 11.70 | [8.50,16.11] |
| 2001 | 2.25 | [2.16, 2.34] | 12.57 | [9.24,17.12] | 3.18 | [3.08, 3.28] | 17.81 | [12.96,24.48] |
| 2002 | 2.40 | [2.31, 2.49] | 13.33 | [9.80,18.13] | 3.22 | [3.13, 3.32] | 18.01 | [13.11,24.74] |
| 2003 | 2.64 | [2.56, 2.72] | 14.58 | [10.72,19.83] | 3.50 | [3.40, 3.59] | 19.47 | [14.18,26.74] |
| 2004 | 2.43 | [2.36, 2.51] | 13.36 | [9.83,18.16] | 2.87 | [2.79, 2.95] | 15.91 | [11.59,21.86] |
| 2005 | 2.20 | [2.13, 2.28] | 12.06 | [8.87,16.40] | 2.63 | [2.56, 2.71] | 14.55 | [10.59,19.97] |
| 2006 | 2.17 | [2.10, 2.24] | 11.88 | [8.74,16.14] | 2.58 | [2.51, 2.66] | 14.23 | [10.36,19.54] |
| 2007 | 2.29 | [2.23, 2.37] | 12.48 | [9.18,16.97] | 2.70 | [2.63, 2.78] | 14.82 | [10.79,20.35] |
| 2008 | 2.11 | [2.04, 2.18] | 11.45 | [8.42,15.56] | 2.57 | [2.50, 2.64] | 14.07 | [10.25,19.32] |
| 2009 | 2.21 | [2.14, 2.27] | 11.92 | [8.77,16.20] | 2.58 | [2.51, 2.65] | 14.07 | [10.24,19.31] |
| 2010 | 2.25 | [2.18, 2.32] | 12.11 | [8.91,16.46] | 2.58 | [2.51, 2.65] | 14.03 | [10.22,19.27] |
| 2011 | 2.46 | [2.39, 2.53] | 13.15 | [9.67,17.87] | 2.82 | [2.74, 2.89] | 15.21 | [11.08,20.89] |
| 2012 | 2.32 | [2.25, 2.38] | 12.29 | [9.04,16.70] | 2.53 | [2.46, 2.60] | 13.62 | [9.92,18.70] |
| 2013 | 2.47 | [2.40, 2.54] | 13.02 | [9.58,17.70] | 2.76 | [2.69, 2.83] | 14.78 | [10.76,20.29] |
| 2014 | 2.50 | [2.43, 2.58] | 13.18 | [9.70,17.92] | 2.78 | [2.71, 2.85] | 14.87 | [10.83,20.42] |
| 2015 | 2.51 | [2.43, 2.59] | 13.08 | [9.62,17.79] | 2.81 | [2.73, 2.89] | 14.94 | [10.87,20.52] |
| 2016 | 2.70 | [2.61, 2.79] | 14.03 | [10.32,19.08] | 3.08 | [2.99, 3.18] | 16.44 | [11.97,22.59] |
| 2017 | 2.67 | [2.58, 2.77] | 13.87 | [10.20,18.88] | 3.06 | [2.96, 3.16] | 16.39 | [11.93,22.53] |
| 2018 | 2.29 | [2.20, 2.38] | 11.82 | [8.68,16.09] | 2.63 | [2.53, 2.72] | 13.98 | [10.17,19.23] |
| (Age band) |  |  |  |  |  |  |  |  |
| 18-39 | 2.28 | [2.25, 2.31] | 1 |  | 1.91 | [1.88, 1.93] | 1 |  |
| 40-59 | 1.85 | [1.83, 1.88] | 0.87 | [0.85,0.89] | 2.10 | [2.08, 2.13] | 1.17 | [1.15,1.20] |
| 60-79 | 2.01 | [1.98, 2.05] | 0.97 | [0.95,0.99] | 2.50 | [2.47, 2.54] | 1.40 | [1.37,1.43] |
| 80-99 | 5.83 | [5.71, 5.95] | 2.77 | [2.69,2.85] | 8.08 | [7.98, 8.18] | 4.43 | [4.32,4.54] |
| (Townsend score) |  |  |  |  |  |  |  |  |
| 1 | 1.36 | [1.34, 1.39] | 1 |  | 1.82 | [1.79, 1.84] | 1 |  |
| 2 | 1.69 | [1.66, 1.72] | 1.22 | [1.18,1.25] | 2.15 | [2.11, 2.18] | 1.15 | [1.12,1.18] |
| 3 | 2.16 | [2.12, 2.19] | 1.57 | [1.52,1.61] | 2.67 | [2.63, 2.71] | 1.46 | [1.42,1.50] |
| 4 | 2.89 | [2.85, 2.93] | 2.10 | [2.04,2.16] | 3.29 | [3.24, 3.34] | 1.80 | [1.75,1.85] |
| 5 | 4.29 | [4.23, 4.36] | 3.18 | [3.09,3.27] | 4.34 | [4.27, 4.40] | 2.46 | [2.40,2.53] |

I – Initiation; aIRR – Adjusted Incidence Rate Ratio.

All rates were adjusted for other characteristics in this table.

1. Unadjusted prevalence of Second-Generation Antipsychotic prescribing (SGA) per 1000 individuals and adjusted Prevalence Rate Ratio (PRR) stratified by sex.

|  | (b) Prevalence | | | | | | | |
| --- | --- | --- | --- | --- | --- | --- | --- | --- |
|  | Male | | | | Female | | | |
| (Year) | P | CI (95%) | aPRR | CI (95%) | P | CI (95%) | aPRR | CI (95%) |
| 1995 | 0.25 | [0.19, 0.33] | 1.00 |  | 0.22 | [0.16, 0.30] | 1.00 |  |
| 1996 | 0.32 | [0.25, 0.40] | 1.28 | [0.89,1.84] | 0.33 | [0.26, 0.40] | 1.47 | [1.03,2.11] |
| 1997 | 0.69 | [0.60, 0.78] | 2.69 | [1.97,3.68] | 0.53 | [0.45, 0.61] | 2.35 | [1.71,3.24] |
| 1998 | 1.12 | [1.02, 1.24] | 4.47 | [3.32,6.01] | 1.05 | [0.95, 1.15] | 4.71 | [3.49,6.37] |
| 1999 | 1.72 | [1.60, 1.84] | 6.81 | [5.09,9.10] | 1.64 | [1.53, 1.75] | 7.37 | [5.50,9.89] |
| 2000 | 2.24 | [2.13, 2.36] | 8.96 | [6.73,11.93] | 2.49 | [2.38, 2.61] | 11.28 | [8.45,15.06] |
| 2001 | 3.24 | [3.13, 3.36] | 12.89 | [9.70,17.13] | 4.11 | [3.98, 4.24] | 18.57 | [13.93,24.74] |
| 2002 | 3.89 | [3.77, 4.01] | 15.44 | [11.63,20.51] | 4.89 | [4.76, 5.02] | 22.04 | [16.55,29.35] |
| 2003 | 4.61 | [4.49, 4.72] | 18.23 | [13.74,24.20] | 5.66 | [5.53, 5.79] | 25.47 | [19.13,33.91] |
| 2004 | 5.05 | [4.93, 5.17] | 19.82 | [14.94,26.30] | 5.87 | [5.75, 5.99] | 26.28 | [19.74,34.98] |
| 2005 | 5.29 | [5.18, 5.40] | 20.63 | [15.55,27.37] | 5.85 | [5.74, 5.97] | 26.05 | [19.57,34.67] |
| 2006 | 5.68 | [5.57, 5.79] | 22.10 | [16.66,29.31] | 6.31 | [6.20, 6.43] | 28.00 | [21.03,37.26] |
| 2007 | 5.93 | [5.81, 6.04] | 23.03 | [17.36,30.55] | 6.72 | [6.60, 6.84] | 29.71 | [22.32,39.55] |
| 2008 | 6.20 | [6.09, 6.31] | 24.02 | [18.10,31.86] | 7.03 | [6.91, 7.15] | 31.01 | [23.30,41.27] |
| 2009 | 6.46 | [6.34, 6.58] | 24.99 | [18.84,33.15] | 7.30 | [7.18, 7.42] | 32.15 | [24.16,42.79] |
| 2010 | 6.86 | [6.74, 6.98] | 26.38 | [19.88,34.98] | 7.63 | [7.51, 7.75] | 33.44 | [25.13,44.50] |
| 2011 | 7.21 | [7.08, 7.33] | 27.59 | [20.80,36.59] | 8.00 | [7.88, 8.13] | 34.89 | [26.22,46.43] |
| 2012 | 7.48 | [7.35, 7.60] | 28.32 | [21.35,37.56] | 8.08 | [7.95, 8.20] | 34.97 | [26.27,46.53] |
| 2013 | 7.82 | [7.69, 7.95] | 29.53 | [22.27,39.17] | 8.43 | [8.30, 8.56] | 36.41 | [27.36,48.45] |
| 2014 | 8.20 | [8.06, 8.34] | 30.94 | [23.33,41.04] | 8.89 | [8.76, 9.03] | 38.31 | [28.79,50.99] |
| 2015 | 8.63 | [8.48, 8.79] | 32.05 | [24.16,42.51] | 9.49 | [9.33, 9.65] | 40.38 | [30.34,53.74] |
| 2016 | 9.40 | [9.22, 9.57] | 34.63 | [26.10,45.93] | 10.37 | [10.19, 10.55] | 44.24 | [33.24,58.88] |
| 2017 | 9.88 | [9.68, 10.07] | 36.42 | [27.45,48.31] | 10.81 | [10.61, 11.01] | 46.20 | [34.71,61.49] |
| 2018 | 10.22 | [10.02, 10.43] | 37.34 | [28.14,49.53] | 11.20 | [11.00, 11.41] | 47.29 | [35.53,62.94] |
| (Age band) |  |  |  |  |  |  |  |  |
| 18-39 | 5.61 | [5.57, 5.66] | 1.00 |  | 4.33 | [4.29, 4.37] | 1.00 |  |
| 40-59 | 7.02 | [6.97, 7.07] | 1.33 | [1.32,1.35] | 7.51 | [7.46, 7.56] | 1.85 | [1.83,1.87] |
| 60-79 | 5.77 | [5.71, 5.82] | 1.13 | [1.12,1.15] | 7.34 | [7.27, 7.40] | 1.83 | [1.81,1.85] |
| 80-99 | 8.72 | [8.57, 8.86] | 1.67 | [1.64,1.70] | 14.69 | [14.55, 14.83] | 3.56 | [3.51,3.61] |
| (Townsend score) |  |  |  |  |  |  |  |  |
| 1 | 3.37 | [3.33, 3.41] | 1.00 |  | 4.52 | [4.48, 4.57] | 1.00 |  |
| 2 | 4.44 | [4.39, 4.49] | 1.29 | [1.27,1.32] | 5.38 | [5.33, 5.44] | 1.16 | [1.14,1.18] |
| 3 | 6.04 | [5.98, 6.10] | 1.75 | [1.72,1.78] | 6.99 | [6.93, 7.05] | 1.53 | [1.51,1.55] |
| 4 | 8.43 | [8.36, 8.51] | 2.46 | [2.42,2.50] | 9.05 | [8.97, 9.13] | 2.02 | [1.99,2.04] |
| 5 | 13.26 | [13.15, 13.38] | 3.89 | [3.83,3.95] | 12.80 | [12.69, 12.92] | 2.95 | [2.91,2.99] |

P – Prevalence; aPRR – Adjusted Prevalence Rate Ratio.

All rates were adjusted for other characteristics in this table.

**Appendix 7.**

(a) Unadjusted initiation of antipsychotic prescribing per 1000 person-years at risk (PYAR),

(b) Unadjusted prevalence of antipsychotic prescribing per 1000 individuals.

**
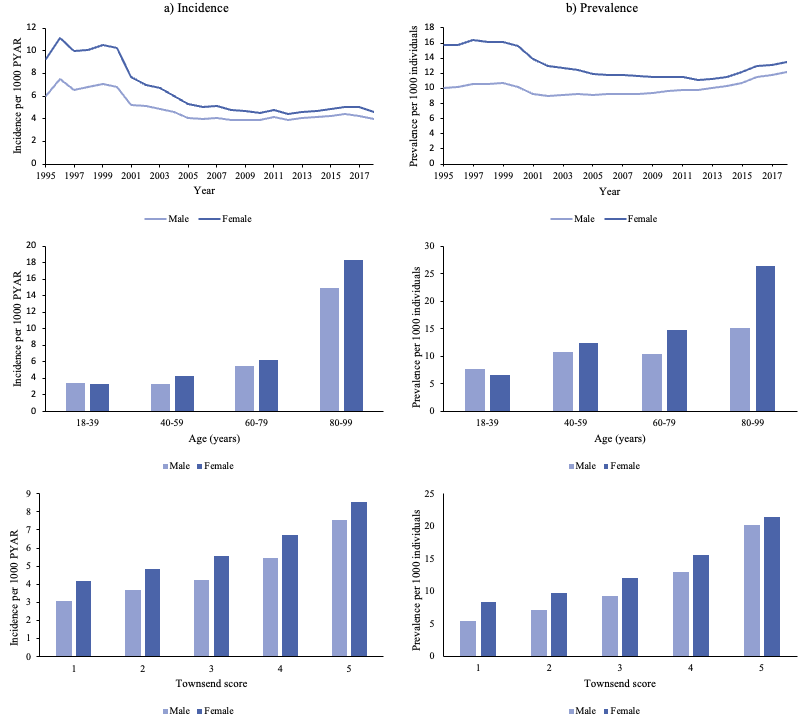
**

**Appendix 8:**

1. Unadjusted initiation of all antipsychotic prescribing by sub-classes from 1995 to 2018, and
2. Unadjusted prevalence of all antipsychotic prescribing by sub-classes from 1995 to 2018.

(a)


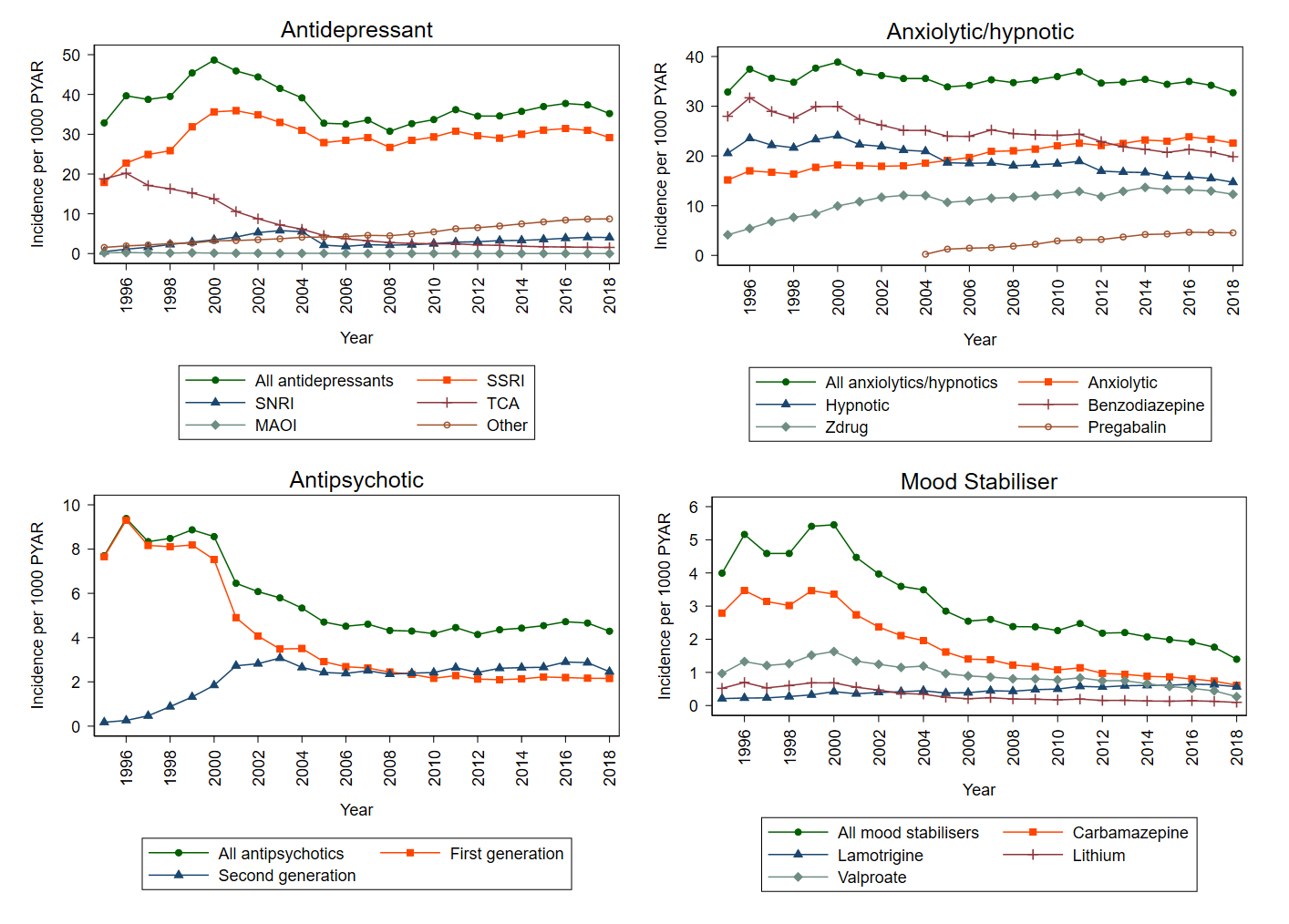


(b)


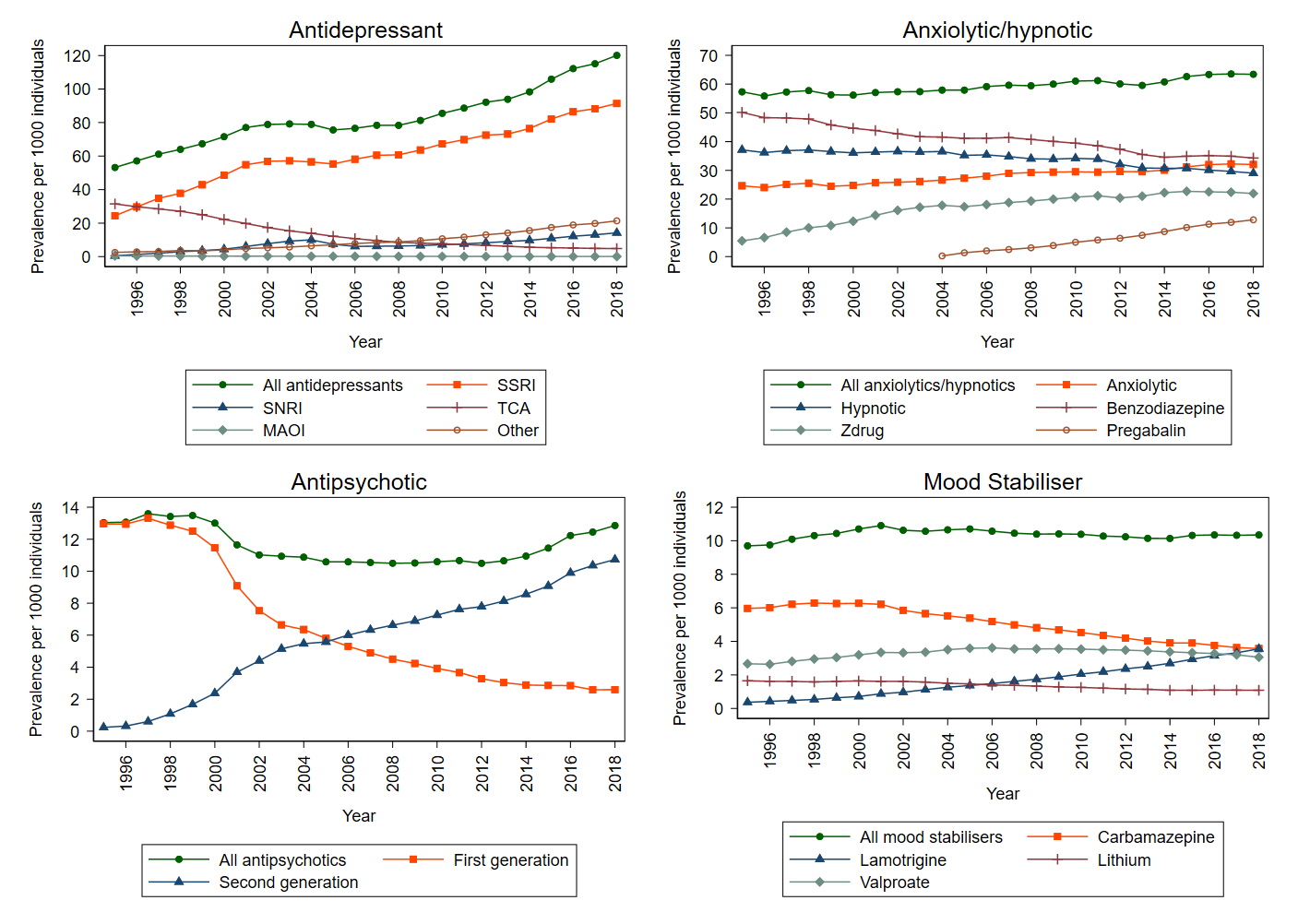

Supplement: Samsuddin et al. supplementary material [file S2056472425108156sup001.docx]
